# Analysis of Genetic Variation in CD40 and CD40L: Relationship with mRNA Relative Expression and Soluble Proteins in Acute Coronary Syndrome

**DOI:** 10.1155/2019/8063983

**Published:** 2019-04-30

**Authors:** D. E. Martínez-Fernández, J. R. Padilla-Gutiérrez, F. Casillas-Muñoz, Emmanuel Valdés-Alvarado, Brenda Parra-Reyna, Maricela Aceves-Ramírez, J. F. Muñoz-Valle, U. Zalapa Flores, J. C. Chávez Herrera, Y. Valle

**Affiliations:** ^1^Instituto de Investigación en Ciencias Biomédicas (IICB), Centro Universitario de Ciencias de la Salud (CUCS), Universidad de Guadalajara (UdeG), Sierra Mojada #950, Colonia Independencia, C.P, 44340 Guadalajara, Jalisco, Mexico; ^2^Doctorado en Ciencias Biomédicas, Centro Universitario de Ciencias de la Salud (CUCS), Universidad de Guadalajara (UdeG), Guadalajara, Jalisco, Mexico; ^3^Doctorado en Genética Humana, Centro Universitario de Ciencias de la Salud (CUCS), Universidad de Guadalajara (UdeG), Guadalajara, Jalisco, Mexico; ^4^Especialidad en Cardiología, Unidad Médica de Alta Especialidad, Centro Médico Nacional de Occidente (CMNO), Departamento de Cardiología, Instituto Mexicano del Seguro Social (IMSS), Guadalajara, Jalisco, Mexico

## Abstract

Acute coronary syndrome (ACS) can be triggered by the presence of inflammatory factors which promote the activation of immune cells by costimulatory molecules such as CD40 and its ligand CD40L. Environmental and genetic factors are involved in the etiology of the ACS. The aim of this study was to explore the gene and protein expression associated with *CD40* and *CD40L* genetic variants in ACS patients from the western Mexican population. A total of 620 individuals from western Mexico were recruited: 320 ACS patients and 300 individuals without a history of ischemic cardiopathy were evaluated. The genotype was determined using TaqMan SNP genotyping assays. *CD40* and *CD40L* expressions at the mRNA level were quantified using TaqMan Gene Expression Assays. Soluble protein isoforms were measured by enzyme-linked immunosorbent assay. We did not find evidence of association between *CD40* (rs1883832, rs4810485, and rs11086998) and *CD40L* (rs3092952 and rs3092920) genetic variants and susceptibility to ACS, although rs1883832 and rs4810485 were significantly associated with high sCD40 plasma levels. Plasma levels of sCD40L can be affected by gender and the clinical spectrum of acute coronary syndrome. Our results do not suggest a functional role of *CD40* and *CD40L* genetic variants in ACS. However, they could reflect the inflammatory process and platelet activation in ACS patients, even when they are under pharmacological therapy. Due to the important roles of the CD40-CD40L system in the pathogenesis of ACS, longitudinal studies are required to determine if soluble levels of CD40 and CD40L could be clinically useful markers of a recurrent cardiovascular event after an ACS.

## 1. Introduction

Cardiovascular disease is the leading cause of death worldwide [1], specifically acute coronary syndrome (ACS) characterized by the acute reduction in coronary blood flow and myocardial ischemia or necrosis. Three clinical entities are distinguished: ST-segment elevation myocardial infarction (STEMI), non-ST segment elevation myocardial infarction (NSTEMI), and unstable angina (UA), with a common etiology; however, they may have different outcomes [2]. For this reason, the early prognosis and risk stratification are the first line of action to reduce the risk of recurrent episodes or even death after an ACS [3].

In the etiology of ACS, metabolic, genetic, environmental, and among other factors are involved, conditions that can increase inflammation and endothelial dysfunction, key elements in the development of atherosclerosis [4, 5]. It has been estimated that the percentage of heritability is 40-55% of the interindividual variation in the risk of ACS supporting the substantial genetic influence [6].

CD40 is a costimulatory molecule that is expressed constitutively in B lymphocytes and expressed in other cells such as monocytes, macrophages, endothelial cells (ECs), and smooth muscle cells (SMCs) [7]. Its ligand, CD40L, is expressed mainly on the surface of platelets (95%), T lymphocytes, ECs, SMCs, and macrophages [7, 8].

The interaction of CD40 and CD40L is involved in the immunopathogenesis of ACS [9] due to its participation in bidirectional cell activation through the signaling pathways c-Jun, NF-*κ*B, and ERK 1/2, promoting the increase of adhesion molecules, secretion of proinflammatory cytokines, and platelet activation [7]. However, the soluble forms of CD40 and CD40L have been associated with endothelial dysfunction or as markers of adverse cardiovascular events in ACS [10, 11] patients suggesting them as potential targets of therapeutic agents [9].

In this regard, single-nucleotide polymorphisms (SNPs) have been identified in the *CD40* and *CD40L* genes that may increase the susceptibility for the development of ACS, through genome-wide association studies [7] and candidate gene studies [12–16]. These genetic variants have been associated with inflammatory and autoimmune diseases [17, 18], with alterations in the *CD40* and *CD40L* mRNA expression [19–25] as well as the regulation of soluble forms modifying their function [15, 20] which have an effect in the initiation, development, and progression of atherosclerosis [26, 27].

Inflammation and endothelial dysfunction are factors that promote the development of atherosclerosis; however, the underlying cellular and molecular interactions, which link the existing risk factors with the atherosclerotic process, are not well characterized. To our knowledge, there are no studies that evaluate the contribution of *CD40* and *CD40L* polymorphisms in the population from western Mexico associated with cardiovascular diseases specifically with ACS.

Accordingly, the aim of this study was to explore the association between single-nucleotide polymorphisms in the *CD40* (rs1883832 (c.-1C>T), rs4810485 (c.51+914G>T), and rs11086998 (c.679C>G)) and *CD40L* (rs3092952 (g.1615A>G) and rs3092920 (18656G>T)) genes and susceptibility to ACS. We also explore the association between these polymorphisms with *CD40* and *CD40L* expression in peripheral blood leukocytes and soluble levels quantified in plasma samples obtained from ACS patients and subjects without ischemic cardiopathy included in a control group (CG).

## 2. Materials and Methods

### 2.1. ACS Patients and Subjects in the Control Group

The study cohort consisted of 320 patients diagnosed with ACS classified according to the American College of Cardiology [28] and 300 subjects with ACS risk factors but no history of ischemic cardiopathy (ascertained by questionnaire) as the CG. All the individuals included in the study from western Mexico were recruited from Hospital de Especialidades del Centro Medico Nacional de Occidente del Instituto Mexicano del Seguro Social (CMNO-IMSS). The period of the study was from February 2016 to June 2018.

### 2.2. Ethical Considerations

The study was conducted in accordance with the 2013 Helsinki Declaration. All individuals agreed to participate in the study and signed informed written consent. The ethical approval was obtained by the Centro Universitario de Ciencias de La Salud (CUCS), UdeG (CI/065/2014).

### 2.3. Genotyping and Quality Control

Genetic variants were genotyping using TaqMan Assays for *CD40* (rs1883832 (C__11655919_20), rs4810485 (C___1260190_10), and rs11086998 (C___1260325_10)) and for *CD40L* (rs3092952 (C__26154899_10) and rs3092920 (C__27452204_10)) using TaqMan Genotyping Master Mix (Applied Biosystems, Foster City, CA, USA), following the manufacturer's protocol. As quality control, a double-blind genotyping of 25% of the samples was performed for all polymorphisms, with no variation in the genotype assignment.

### 2.4. Real-Time PCR Analysis

Total RNA was extracted from peripheral blood leukocytes using TRIzol reagent (Invitrogen, Carlsbad, CA, USA) according to the manufacturer's instruction to obtain total RNA according to the method of Chomczynski and Sacchi [29]. One microgram of total RNA was reverse transcribed using reverse transcription reagents to obtain cDNA following the manufacturer's protocol (Promega Corporation, USA). Real-time PCR was performed using TaqMan probes: glyceraldehyde 3-phosphate dehydrogenase (*GAPDH*) (Hs02786624_g1), *CD40* (Hs01002915_g1), and *CD40L* (Hs00163934_m1) according to the conditions indicated in TaqMan Gene Expression Assay Protocol (Applied Biosystems, Foster City, CA, USA) using a Roche LightCycler 96® System. Relative expression was normalized to the expression level using *GAPDH* as an internal control and was evaluated using the 2^-ΔΔCq^ and 2^-ΔCq^ methods [30]. Results are expressed as a relative fold increase compared with control and unit relative of expression (URE), respectively. The 2^-∆∆Cq^ method was calculated as is suggested by Livak and Schmittgen [30]; the aim of this method is to compare the expression measures of genes of interests through the normalization to a reference gene in this case GAPDH. Using the following equation: *∆*Cq = (average Cq of the gene of interest–average Cq of reference gene), we calculated *∆∆*Cq = (*∆*Cq–*∆*Cq calibrator), where ∆Cq calibrator refers to the average ∆Cq of the control group. We determined the relative CD40 mRNA expression in peripheral blood leukocytes from ACS patients (*n* = 42) and control group (*n* = 18). For the assessment of the expression levels, cohort characteristics were as follows: age (62 ± 8), gender (female/male ratio; ACS: 1.7, CG: 1.6), and similar risk factor presence, and the same number of patients was included for each of the three clinical entities of ACS (14 UA, 14 NSTEMI, and 14 STEMI).

### 2.5. Detection of sCD40, sCD40L, and IFN-*γ*

The levels of sCD40, sCD40L, and IFN-*γ* were determined on plasma samples using enzyme-linked immunosorbent assays (ELISA) (Human CD40 Quantikine ELISA Kit, Human CD40 Ligand Quantikine, and Human IFN-gamma Quantikine ELISA Kit, R&D Systems) according to the manufacturer's protocol. The assay sensitivity for sCD40, sCD40L, and IFN-*γ* was 5.56 pg/mL, 10.1 pg/mL, and 8 pg/mL, respectively.

### 2.6. Statistical Analysis

Statistical analysis was performed using SPSS software version 22 (SPSS Inc., Chicago, IL, USA). Data were presented as a median and interquartile range (IQR) unless otherwise indicated. The Mann-Whitney *U* test and Kruskal-Wallis test were used to compare differences between groups. Genotype and allele frequencies of *CD40* and *CD40L* genetic variants were compared between the ACS patients and control group. Odds ratios (OR) and 95% confidence intervals (CIs) were calculated using Chi-squared and Fisher's exact tests when appropriate. Bonferroni's correction was applied to reduce the statistical type 1 error (pc) in multiple comparisons. Hardy-Weinberg equilibrium was calculated from genotype distribution, and the linkage disequilibrium (LD) between the polymorphisms was calculated using Lewontin's *D*′ and *r*^2^ between genetic markers. The haplotypes and their frequencies were estimated using the SHEsis software platform (http://analysis.bio-x.cn/myAnalysis.php). A multilinear regression model was used to estimate the association of common risk factors (comorbidities, gender, medication intake, etc.) with sCD40, sCD40L, and mRNA expression levels. Correlation analysis was ascertained by Spearman's correlation.

## 3. Results

### 3.1. Clinical Features

The clinical information of the 320 ACS patients and 300 control subjects was recorded in Table 1. ACS patients and CG subjects had a mean age of 62 (±11 standard deviation (SD)) and 55 (±10 SD) years, respectively. In the ACS group, there are 3.32 more males than females; while in the CG, the male/females ratio was almost 1/1. ACS patients presented high levels of cardiac function markers including CK, CK-MB, and troponin I with respect to the reference values; the biochemical parameters are significantly elevated in ACS patients compared to control as shown in Table 1. The risk factors with the highest prevalence in ACS were high blood pressure, smoking, and diabetes mellitus type 2. Reinfarction was found in 49 cases (15.3%). The medication intake included antiplatelet (acetylsalicylic acid, clopidogrel), statins, anticoagulants (heparin and enoxaparin), and antihypertensive agents (captopril, enalapril, spironolactone, and furosemide).

### 3.2. Genotype and Allele Analysis

The genotype and allele frequencies of the *CD40* (rs1883832, rs4810485, and rs11086998) and *CD40L* (rs3092952 and rs3092920) polymorphisms are shown in Tables 2–4. Genotype distributions agreed with Hardy-Weinberg equilibrium expectations (*p* > 0.05) for all SNPs.

Neither genotype nor allele distribution of three *CD40* polymorphisms was statistically different when the ACS and control groups were compared.

Because *CD40L* is located on the X chromosome, we performed the analysis by gender (Table 3). It is noteworthy to mention that we did not find differences for allele frequencies on *CD40L* variants between genders (rs3092952 A/G: *p* = 0.73; rs3092920 G/T: *p* = 0.96). Genetic distribution of both polymorphisms was similar between patients and controls either pooled (data not shown) or gender separated.

### 3.3. Haplotype Analysis of *CD40* and *CD40L*

We analyzed the association between haplotypes found in our western Mexican cohort of the CD40/CD40L genes and ACS. Analysis showed that rs1883832, rs4810485, and rs11086998 of *CD40* were in linkage disequilibrium (*D*′ = 0.83, *r*^2^ = 0.021, *p* = 0.010). We found one major haplotype (CCG) that accounted for 67.1% and 65.5% in ACS patients and control subjects, respectively. We also found a protective haplotype (CTC, OR: 0.22; *p* = 0.0001) (Table 4).

*CD40L* polymorphisms were not in LD (*p* = 0.756), indicating 3092952 and rs3092920 need to be analyzed separately.

### 3.4. *CD40* mRNA Expression

ACS patients exhibited higher median expression of *CD40* mRNA in peripheral blood leukocytes compared to controls (0.82-fold more); however, this comparison was not significant. Similarly, stratification by clinical diagnosis in the ACS group (UA, NSTEMI, and STEMI) indicated no differences in the level of mRNA expression of *CD40*. Next, we analyzed the data from the ACS patients and CG stratifying the results by carrying rs1883832, rs4810485, and rs11086998 genotypes. Because of the low frequencies of minor alleles, we assumed the dominant model of association (rs1883832: C/C vs. C/T+TT, r4810485: G/G vs. G/T+TT, and rs11086998: C/C vs. C/G+G/G), although we did not find differences in the level of *CD40* expression between genetic variants in either group (data not shown).

### 3.5. *CD40L* mRNA Expression

**Figure 1:**
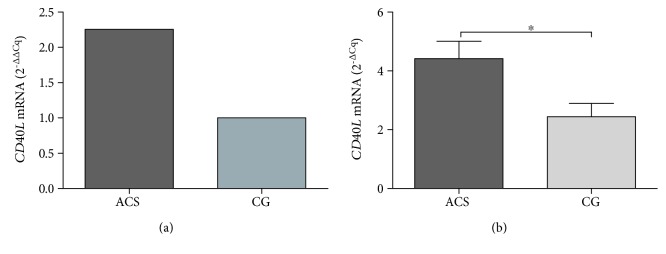
Comparison of *CD40L* mRNA expression in the ACS and CG. Data were analyzed by the 2^-ΔΔCq^ and 2^-ΔCq^ methods normalized to *GAPDH*. CD40 mRNA expression in ACS patients (*n* = 42) and CG (*n* = 18). ACS: acute coronary syndrome; CG: control group. ^∗^*p* < 0.05 by the Mann-Whitney *U* test.

Analysis by the 2^-ΔΔCq^ method showed that *CD40L* mRNA expression in ACS patients was 1.25-fold higher than that in the CG (Figure 1(a)); this difference was significantly ascertained by the 2^-ΔCq^ method (*p=*0.035) (Figure 1(b)). By clinical entities, we did not find any difference (data not shown).

**Figure 2:**
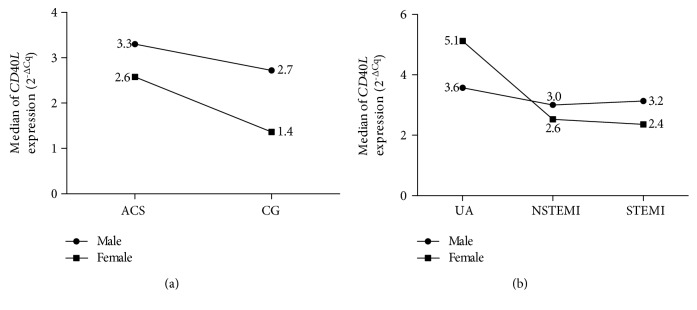
Comparison of *CD40L* mRNA expression in ACS patients between genders. Data were analyzed by the 2^-ΔCq^ normalized to *GAPDH. CD40L* mRNA expression and gender. ACS: acute coronary syndrome; CG: control group, UA: unstable angina; NSTEMI: non-ST-segment elevation myocardial infarction; STEMI: ST-segment elevation myocardial infarction.

Given the location on the X chromosome and the conclusion of other studies that *CD40L* mRNA expression can be affected by gender [20], we analyzed separately for each gender in both groups of study without any evidence of association. By gender, either female or male patients tended to show more expression compared to the control group (Figure 2(a)). When we stratified by clinical diagnosis of ACS and gender, UA female patients showed the highest expression (5.12 URE), although none of the comparisons were statistically significant (male patients, *p* = 0.93; female patients, *p* = 0.53) (Figure 2(b)).

It is important to mention that we explored the *CD40L mRNA* expression in both groups of study according to genetic variants (rs3092952 and rs3092920), following the dominant model; however, we did not find differences.

### 3.6. Association between sCD40 and sCD40L with ACS

We evaluated plasma levels of CD40 and CD40L in both study groups. We found that ACS patients present significantly higher levels of sCD40 (*p* > 0.001) compared with the control group (843.3 vs. 492 pg/mL). A multilinear regression model denotes that this association could be masked by clopidogrel (*p* = 0.005). ACS diagnosis seems not to be associated with sCD40 (UA: 677.1 pg/mL; NSTEMI: 661.7 pg/mL; and STEMI: 782.4 pg/mL, *p* = 0.44).

**Figure 3:**
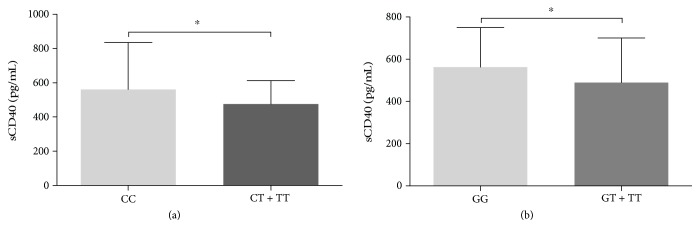
Soluble levels of CD40 are associated with rs1883832 and rs4810485 in control individuals. Association between sCD40 levels and rs1883832 (a) and rs4810485 (b) in control subjects (*n* = 168). ^∗^*p* < 0.05 by the Mann-Whitney *U* test.

Differences between sCD40 levels among CD40 genetic variants were also analyzed assuming the dominant model of association. As shown in Figure 3, C/C carriers had higher plasma sCD40 levels than rs1883832 C/T+T/T (561 vs. 475.7 pg/mL, *p* = 0.005). Similarly, homozygous carriers of rs4810485 showed a higher concentration of soluble CD40 than G/T+T/T carriers (562.5 vs. 489.7 pg/mL, *p* = 0.015).

**Figure 4:**
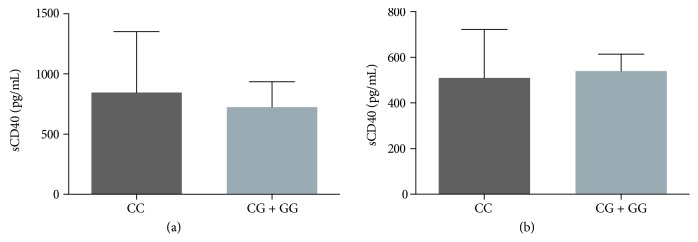
Soluble levels of CD40 and rs11086998 in ACS patients and control individuals. Association between sCD40 levels and rs11086998 in ACS patients (a) and in control subjects (b) (*n* = 168, for each group). ^∗^*p* < 0.05 by the Mann-Whitney *U* test.

In ACS patients, there was no such association of these CD40 genetic variants nor an association of the *CD40* rs11086998 polymorphism with CD40 plasma levels in both groups of study assuming the dominant model of association C/C vs. C/G+G/G (ACS: 846 pg/mL vs. 725 pg/mL, *p* = 0.318; CG: 509 pg/mL vs. 540 pg/mL, *p* = 0.195) (Figure 4).

**Figure 5:**
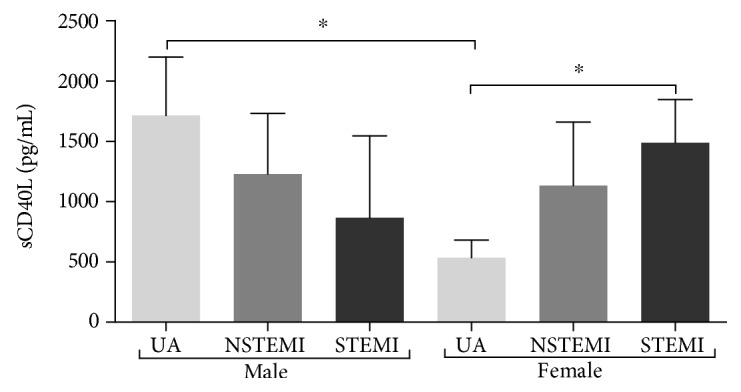
Soluble levels of CD40L in ACS patients between genders. Association between sCD40L between males (*n* = 136) and females (*n* = 32) in ACS patients (*n* = 168). ^∗^*p* < 0.05 by the Mann-Whitney *U* test.

Regarding sCD40L plasma levels, patients exhibit higher levels of sCD40L compared with controls; however, this comparison did not reach statistical significance (1080 vs. 868.9 pg/mL, *p* = 0.16). According to ACS diagnosis and sCD40L stratified by gender, female patients with STEMI had higher levels compared with UA female patients (1489.5 vs. 533.8 pg/mL, *p* = 0.026). UA male patients presented higher levels of sCD40L compared to UA female patients (1714.3 vs. 533.8 pg/mL, *p* = 0.016, Figure 5). When pooled, the levels of sCD40L were similar by ACS diagnosis (*p* = 0.75, data not shown).

Medication did not have a significant effect on plasma sCD40L in the ACS group when introduced in the multiregression model (*β*: 0.003, *p* = 0.98); i.e., differences between patients with ACS of sCD40L are not driven by medication.

We analyzed the association between genetic variants on *CD40L* and plasma levels of sCD40L in both groups of study without any evidence of association.

**Figure 6:**
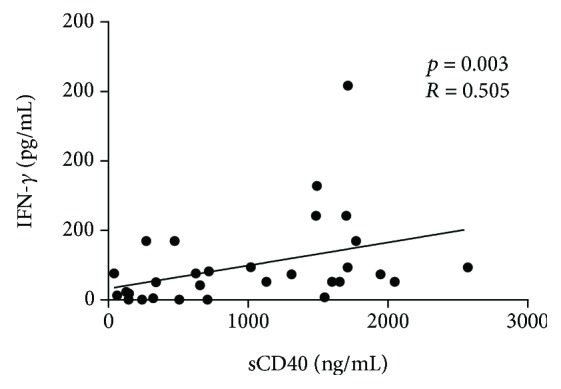
Correlation between sCD40L and IFN-*γ* in ACS patients. Correlation was analyzed with Spearman's correlation coefficient (*r*) and *p* value.

Finally, we observed a positive linear correlation between sCD40L and IFN-*γ* in ACS patients (*r* = 0.505, *p* = 0.003) (Figure 6).

## 4. Discussion

CD40 and CD40L have important functions that comprise cellular activation and production of proinflammatory cytokines. This promotes inflammation that can accelerate atherosclerosis processes and have a functional role in the development of cardiovascular diseases [8]. Therefore, genetic variants have been of interest for their relationship with the increased risk of cardiovascular diseases [14, 15].

The SNP on the *CD40* gene, rs1883832, has been associated with ACS in the presence of the major C allele and has been related with decreased levels of sCD40 in the presence of the minor T allele and risk of cardiovascular diseases [31, 32]. In a western Mexican cohort, this was not identified as a genetic risk factor to rheumatoid arthritis (RA) [25]. This polymorphism has been associated with diminished mRNA expression of CD40 [21, 22]; rs1883832 has been found in high LD with rs4810485, the latter being significantly associated with coronary artery lesion formation in a Taiwanese population [33]. Given the localization on region 5′UTR and intron 1 of these SNPs, respectively, they have been associated with changes in mRNA expression because they are in almost complete LD (*r*^2^ = 0.976) with rs6074022, which resides within the CD40 promoter and affects levels of mRNA [20]. Otherwise, to the best of our knowledge, rs11086998 has not been associated as a genetic risk factor in cardiovascular diseases, but it is known to be related in the modulation of the levels of proinflammatory cytokines, TNF*α* and IL-6, because it resides on exon 9 encoding the intracellular domain protein implicated in signaling pathways of CD40 [34].

These polymorphisms have been previously identified as genetic markers of cardiovascular disease mainly in the Chinese and European population: Li et al. studied 160 ACS patients and 92 controls in a Chinese Han population [12]; Tian et al. analyzed 248 ACS patients and 206 controls in a Chinese population [13]; Wang et al. reported the analysis in 474 patients with coronary atherosclerotic plaques and 225 controls in a Chinese population [14]; and García-Bermúdez et al. analyzed 290 patients with rheumatoid arthritis (RA) with cardiovascular events and 1285 patients with RA but without cardiovascular events [17]. However, in our study, we cannot support this association. This could be explained by genetic differences among populations [35]. The three variants on CD40 were found in LD which means they need to be analyzed together at least in our population. In this context, we found that the haplotype block rs1883832 (C)-rs4810485 (T)-rs11086998 (C) is associated with a decreased risk of ACS (OR: 0.22; *p* = 0.0001) and it may be a protective factor of ACS in the western Mexican population, although it cannot be ruled out that other nearby SNPs in the LD block are associated with disease susceptibility [21].

The analysis of genetic variation in the X chromosome in diseases is an area of interest. In line with the above, in *CD40L*, we evaluated two SNPs located in the 5′UTR (rs3092952) and 3′UTR (rs3092920), they are located in different haplotype blocks and have a weak LD [36] as we corroborated. rs3092952 has been described as a functional variant by modulating the levels of sCD40L in plasma samples. While rs3092920 has been studied as a genetic marker of cardiovascular disease in RA patients and myocardial infarction, some studies did not find such association [15, 17], which is consistent with our results, because we did not find an association between genetic variants on the *CD40L* gene and risk of ACS.

Notwithstanding, other factors may increase the risk of ACS and influence adverse events [5]; our results do not show a significant role of these polymorphisms in acute coronary events. However, the cellular and molecular mechanisms that underlie the effect of these genetic variants and ACS could potentially be mediated by changes in the gene and protein expression of CD40 and CD40L.

Other studies analyzed *CD40*/*CD40L* mRNA expression levels in coronary artery disease (CHD), following the separation of peripheral blood mononuclear cells and qRT-PCR, and found significantly increased expression levels of both genes in the CHD group compared to the control group [24].

We did not find differences between *CD40* mRNA levels in total peripheral blood leucocytes of ACS patients and controls subjects. This may be due to the fact that the main source of CD40 in the peripheral blood is B cells [21]. Field et al. did not find an association between the genotype and CD40 mRNA expression measured in whole blood; however, the C/C rs1883832 genotype was associated with higher CD40 expression in B cells [21]. We suggest that further studies that analyze these subpopulations need to be carried out to understand the functionality of these genetic variants in CD40 mRNA gene expression.

The lack of association between genetic polymorphisms and the CD40 expression agrees with that reported by Jacobson et al. [22], since they suggest that the rs1883832 genetic variant, which is in high linkage disequilibrium to rs4810485 (DL = 0.83), acts at the level of translation rather than transcription, exerting its effects as a functional polymorphism in the etiology of disease through the regulation of efficiency of translation, in the absence of any genotype effect on mRNA levels. In the same line, the rs4810485 SNP was associated with CD40 protein expression on CD14^+^ monocytes and CD19^+^ B cells from lupus erythematosus patients assessed by flow cytometry [18]. Other studies have reported increased levels of sCD40 in subjects who carry the minor allele of rs1883832 and rs4810485 polymorphisms [25, 32].

We observed increased levels of sCD40 in plasma samples from acute coronary patients compared to the control group (*p* > 0.001). During inflammatory processes, sCD40 has biological properties described as immunomodulatory, because it inhibits immune responses [37]. The proinflammatory systemic response has a key role in the pathophysiology of this syndrome contributing to the cardiovascular outcome; in this sense, sCD40 represents a potential element by which the CD40-CD40L system can be modulated through its antagonistic effect against CD40 activation [38].

The clinical entities of ACS did not differ in plasma sCD40 levels (*p* = 0.44) even when they have different outcomes, and sCD40 is involved in several stages of development of ACS [39]. We highlight that our results need to be taken with caution since clopidogrel explained 25% of the total variability of CD40 plasma levels in ACS and increased concentrations of blood lipids appeared as significant predictors of sCD40 levels (denoted by linear regression, *p* = 0.027). Decreased cholesterol synthesis by pharmacological therapy used in ACS patients has been associated with increased levels of sCD40 [40]. It is important to clarify that clopidogrel is one of the drugs that constitute the cornerstone in the treatment scheme of these patients, so this is an intervening variable that should be considered in future studies. Unfortunately, the number of patients that is not treated with clopidogrel is quite small hampering robust comparisons.

Pharmacological therapy can have an effect on plasma levels of CD40 [41], a circumstance that would explain the lack of association between the polymorphisms on *CD40* locus and the soluble levels of plasma samples in ACS patients. An argument in favor of this is the findings in the control group; we found that homozygous carriers of the major allele of rs1883832 and rs4810485 had significantly higher levels of sCD40 (Figure 3).

Patients with ACS had increased level of *CD40L* mRNA compared to controls. Importantly, elevated levels of *CD40L* mRNA expression on T lymphocytes are related to acute coronary events [42]. Although we did not find significant differences between ACS and CG in sCD40L plasma levels, we observed that patients had higher levels of sCD40L and a positive linear correlation between sCD40L and IFN-*γ* was found (Figure 6); thus, it may be speculated that acute coronary event patients have enhanced T cell responses promoting endothelial dysfunction and systemic inflammation [43, 44].

A previous report has concluded that *CD40L* mRNA expression can be affected by gender [20]; in addition, a decreased level of CD40L mRNA and increase sCD40L concentrations could reflect greater platelet activity that supports CD40L function in the immunopathology of ACS [7]. Accordingly, we only found a higher plasma sCD40L concentration in ACS patients. By clinical entities, females with UA had a lower concentration than those with STEMI; the opposite effect was seen in males. Noteworthy, the three clinical spectrums of ACS have often the same symptomatology [45] and the scientific community is urged to find biomarkers that complement the diagnosis.

Moreover, it has been reported that 4% to 30% of patients with cardiovascular ischemic diseases have poor response to antiplatelet therapy labeled as “low responders” or “nonresponders” [46]; specifically, male patients have been reported to be 2.9 times more likely to be nonresponders to antiplatelet therapy than females [47] and have been linked to recurrent cardiovascular event as death due to coronary heart disease, a major nonfatal coronary event (myocardial infarction, hospitalization for unstable angina, or resuscitated cardiac arrest), or fatal ischemic stroke [48].

The persistence of platelet reactivity even with the use of antiplatelet agents has aroused interest in strategies capable of identifying subjects with a high risk of recurrent cardiovascular events after an ACS. In this regard, plasma concentration of CD40L had been associated as a marker of myocardial damage and independently predicts death and recurrent MI in patients with ACS [10]. Hence, the adjustment of the antiplatelet therapy used in ACS according to gender and risk factors could reduce the risk of adverse events and improve the outcome of patients.

Nevertheless, we have in mind that our results should be interpreted with caution and replicated because of the sample size effect under stratified analysis.

Finally, the analysis of the association of the two genetic variants of CD40L is not correlated with mRNA expression and protein concentration. Further studies are required considering modifiable factors that can change gene expression to understand the functional effect of these polymorphisms in CD40 and CD40L expression.

## 5. Conclusions

Individually analyzed, genetic variants of *CD40* and *CD40L* are not susceptibility markers of ACS in this Mexican cohort. However, the CTC haplotype of CD40 was associated with decreased risk of ACS.

ACS patients exhibited higher *CD40L* mRNA relative expression than controls.

The soluble levels of CD40 were elevated in ACS patients; however, they are affected by clopidogrel therapy.

In controls, rs1883232 C/C and rs4810485 G/G genotype carriers showed higher soluble levels of CD40.

Female patients with STEMI had higher levels of sCD40L compared with UA. Otherwise, UA male patients presented higher levels of sCD40L compared to UA. Medication did not have a significant effect on plasma sCD40L.

These results could reflect the inflammatory process and platelet activation in ACS patients, even when they are under antiplatelet and anti-inflammatory therapy. Due to the important roles of the CD40-CD40L system in immunologic processes in the pathogenesis of ACS, longitudinal studies are required to determine if soluble levels of CD40 and CD40L could be clinically useful markers of a recurrent cardiovascular event after an ACS.

## Figures and Tables

**(a):** 

Variable	ACS median (IQR 25-75)	CG median (IQR 25-75)	Reference values	*p*
Age (years)	62 ± 11^a^	55 ± 10^a^	—	<0.001
Male/female ratio	3.32	1.05	—	<0.001
Cholesterol total (mg/dL)	115 (93-139)	162 (138-197)	150-199	<0.001
Glucose (mg/dL)	125 (97-171)	94 (82-118)	75-105	<0.001
Triglycerides (mg/dL)	89 (72-108)	99 (80-133)	<200	<0.001
LDL-c (mg/dL)	40 (33-52)	68 (51-95)	<130	<0.001
HDL-c (mg/dL)	19 (13-26)	37 (23-54)	>40	<0.001
CRP (mg/L)	19 (3.3-34.9)	9.4 (8.2-11.8)	1-10^∗^	<0.001
Apo A-I (mg/dL)	166 (148-184)	197 (179-213)	94-178	<0.001
Apo B (mg/dL)	132 (109-155)	166 (147-186)	63-133	<0.001
CK (IU/mL)	361 (147-1023)	N.A.	24-195	—
CK-MB (IU/mL)	48 (22-131)	N.A.	<12	—
Troponin I (ng/mL)	3 (0.57-7.1)	N.A.	0.1-0.4	—

**(b):** 

Risk factor	ACS *n* (%)	CG *n* (%)	*p*
Obesity	87 (27.2)	35 (11.1)	<0.001
Diabetes mellitus type 2	148 (46.3)	59 (18.7)	<0.001
Dyslipidemia	140 (43.8)	45 (14.2)	<0.001
High blood pressure	214 (66.9)	90 (28.5)	<0.001
Smoking	159 (49.7)	43 (13.6)	<0.001

**(c):** 

ACS diagnosis	*n* (%)	ACS treatment	*n* (%)
UA	38 (11.9)	Acetyl salicylic acid	304 (95)
NSTEMI	64 (20)	Statins	291 (91)
STEMI	218 (68.1)	Antiplaquetary agents	289 (90.3)
		Antihypertensive agents	180 (56.2)
		Anticoagulants agents	219 (68.3)
		Beta-blockers	181 (56.6)
		Nitrates (isosorbide)	69 (21.7)

^∗^Mann-Whitney *U* test; ACS: acute coronary syndrome; Apo A-I: apolipoprotein A-I; Apo B: apolipoprotein B; CG: control group; CK: creatinine kinase; CK-MB: creatinine kinase muscle and brain; CRP: C-reactive protein; HDL-c: high-density lipoprotein; IQR: interquartile range; LDL-c: low-density lipoprotein; NSTEMI: non-ST-segment elevation myocardial infarction; N.A.: not applicable; UA: unstable angina; STEMI: ST-segment elevation myocardial infarction (STEMI). The data were expressed as a median and interquartile range (Q25-Q75) unless otherwise indicated. ^a^Data provided in mean ± SD. ^∗^Age-dependent.

**Table 2:** Allele and genotype frequencies of the CD40 rs1883832, rs4810485, and rs11086998 in the ACS and CG.

	ACS *n* (%)	CG *n* (%)	OR (CI 95%)	*p*
rs1883832				
*Genotype*				
C/C	188 (58.8)	194 (64.7)	1	—
C/T	121 (37.8)	95 (31.7)	1.31 (0.94-1.84)	0.10
T/T	11 (3.4)	11 (3.6)	1.03 (0.44-2.44)	0.94
*Allele*				
C	497 (77.7)	483 (80.5)	1.19 (0.90-1.56)	0.22
T	143 (22.3)	117 (19.5)		

rs4810485				
*Genotype*				
G/G	186 (58.1)	173 (57.6)	1	—
G/T	118 (36.9)	113 (37.7)	0.97 (0.70-1.35)	0.86
T/T	16 (5)	14 (4.7)	1.06 (0.50-2.24)	0.87
*Allele*				
G	490 (76.6)	459 (76.5)	0.99 (0.77-1.30)	0.97
T	150 (23.4)	141 (23.5)		

rs11086998				
*Genotype*				
C/C	268 (83.8)	242 (80.7)	1	-
C/G	47 (14.7)	52 (17.3)	0.82 (0.53-1.26)	0.36
G/G	5 (1.5)	6 (2)	0.75 (0.23-2.50)	0.64
*Allele*				
C	583 (91)	536 (89.3)	0.82 (0.56-1.19)	0.29
G	57 (09)	64 (10.7)		

ACS: acute coronary syndrome; CG: control group; CI: confidence interval; OR: odds ratio.

**Table 3:** Genotype and allele frequency of the *CD40L* rs3092952 and rs3092920 in the ACS and CG by gender.

	ACS *n* (%)	CG *n* (%)	OR (CI 95%)	*p*		*p* ^a^
rs3092952					MAF	
*Allele*	Male					
A	115 (46.7)	89 (54.9)	1.39 (0.93-2.07)	0.11	0.50	0.73
G	131 (53.3)	73 (45.1)				
*Allele*	Female					
A	68 (45.9)	154 (50)	1.18 (0.79-1.74)	0.42	0.51	
G	80 (54.1)	154 (50)				
*Genotype*	Female					
A/A	18 (24.3)	37 (24)	1	-		
A/G	32 (43.2)	80 (52)	0.82 (0.41-1.65)	0.58	-	-
G/G	24 (32.5)	37 (24)	1.33 (0.62-2.86)	0.46		

rs3092920					MAF	
*Allele*	Male					
G	197 (80)	138 (85.2)	1.43 (0.84-2.44)	0.19	0.180	0.96
T	49 (20)	24 (14.8)				
*Allele*	Female					
G	120 (81)	253 (82.1)	0.93 (0.56-1.54)	0.78	0.182	
T	28 (19)	55 (17.9)				
*Genotype*	Female					
G/G	48 (64.9)	104 (67.5)	1	-	-	-
G/T	24 (32.4)	45 (29.2)	0.86 (0.47-1.58)	0.64		
T/T	2 (2.7)	5 (3.3)	1.15 (0.22-6.16)	0.87		

ACS: acute coronary syndrome; CG: control group; CI: confidence interval; MAF: minor allele frequencies; OR: odds ratio. ^a^*p* value for the allelic model between genders.

**Table 4:** CD40 haplotype distribution in the ACS patients and control group.

*CD40* haplotype	ACS 2*n* (%)	CG 2*n* (%)	OR (CI 95%)	*p*
CCG	403 (67.1)	386 (65.5)	1	—
CGG	58 (9.7)	65 (9.8)	0.85	0.42
CTC	7 (1.2)	30 (5.1)	0.22	0.0001
TTC	132 (22)	108 (18.3)	1.17	0.29

ACS: acute coronary syndrome; CG: control group; CI: confidence interval; corrected significance level (pc) < 0.016; OR: odds ratio. Haplotype is represented by rs1883832, rs4810485, and rs11086998, respectively.

## Data Availability

The data used to support the findings of this study are included in the article.
